# *EGFR*敏感突变的NSCLC脑转移患者治疗模式之争：临床到底该如何选择？

**DOI:** 10.3779/j.issn.1009-3419.2020.101.29

**Published:** 2020-08-20

**Authors:** 程 程, 洪卿 庄

**Affiliations:** 100191 北京, 北京大学第三医院肿瘤放疗科 Department of Radiation Oncology, Peking University Third Hospital, Beijing 100191, China

**Keywords:** 脑转移, 表皮生长因子受体, 肺肿瘤, 放疗, Brain metastasis, Epidermal growth factor receptor, Lung neoplasms, Radiotherapy

## Abstract

表皮生长因子受体(epidermal growth factor receptor, *EGFR*)敏感突变的脑转移是靶向时代非小细胞肺癌(non-small cell lung cancer, NSCLC)治疗的热点和难点, 也是当前肺癌治疗领域争论的焦点。因不同研究的不同结果以及不同学科的理解差异, 这一治疗领域一直伴随着不同的声音, 肿瘤内科的主导模式为没有症状的患者可以先用靶向治疗, 等出现症状或者疾病进展后再行局部放疗, 即以症状和进展作为局部治疗介入的指征和标准, 没有症状的情况下, 给予局部放疗, 有可能增加患者的痛苦, 属于过度治疗, 而放疗学科视角则认为脑转移需要尽早处理, 否则可能影响患者生存。尽早治疗局部病变, 增加治疗深度, 有助于达到延长患者生存时间的目的。学科之争给临床医生选择带来诸多困惑, 本文参考相关文献, 从追寻治疗疾病真相和解决问题的角度总结与讨论, 以期为临床实践提供参考。

## Abstract (en)

Brain metastasis of epidermal growth factor receptor (*EGFR*) sensitive mutations is a hot and difficult point in targeted era of non-small cell lung cancer (NSCLC) treatment, meanwhile it is also the central issue of controversy in the field of lung cancer treatment. Different results of different studies and different understanding of different disciplines, this field of treatment has been accompanied by different voices, patients without clinical symptoms can use targeted therapy first, and then start local radiotherapy with clinical symptoms or disease progression. It is a major model of the medical oncology. That is to say, taking symptoms and progress as indication and standard of local treatment intervention. In the absence of symptoms, local radiotherapy may increase patients' pain, which belongs to overtreatment. However, the perspective of radiotherapy is that brain metastases need to be treated clinically as early as possible, if not, it may affect the survival of patients. Early treatment of local lesions and increasing the depth of treatment are helpful to prolong the survival time of patients. This article refers to relevant literatures and summarizes the discussion from the perspective of pursuing the truth of disease treatment and problem solving in order to provide reference for patients' clinical practice.

十几年来, 肺癌靶向治疗是肺癌治疗领域的主要突破, 肺癌研究取得了令人欣喜的成绩, 表皮生长因子受体(epidermal growth factor receptor, *EGFR*)敏感突变的非小细胞肺癌(non-small cell lung cancer, NSCLC)患者生存期明显延长, 患者生存期从化疗时代的8个月-11个月延长到25个月-39个月左右^[1-9]^。患者生存期的延长也使更多患者具备脑转移的可能, *EGFR*敏感突变的NSCLC脑转移患者成为近十年来这一领域的难点和热点。同时, 因为不同研究的不同结果以及不同学科的理解差异, 这一治疗领域一直伴随着不同的声音, 特别是肿瘤内科和肿瘤放疗学科的争论, 也引起了临床医生在治疗实践中的众多困惑。本文总结相关文献, 结合作者自己的观点, 以飨读者, 以期为临床治疗提供参考。

## 1 当前什么治疗模式？

### 1.1 当前内科推荐模式

从一代EGFR酪氨酸激酶抑制剂(tyrosine kinase inhibitor, TKI)开始, 肺癌脑转移就是治疗的热点, 无可否认的是, TKI药物与传统化疗相比, 其因为分子量小、脂水比例优势、渗透性好的优势^[10-13]^, 在脑转移中取得了较传统治疗令人振奋的效果, 对*EGFR*敏感突变脑转移治疗产生了巨大推动作用。早期有很多尝试性I期-II期或者回顾性研究^[14-17]^, 但真正奠定这一治疗模式的是吴一龙教授的CTONG0803研究^[18]^, 该研究通过对*EGFR*敏感突变和非突变患者行TKI治疗, 结果显示单药使用厄洛替尼对于NSCLC伴有脑转移患者具有良好的疗效和耐受性, 同时*EGFR*敏感突变脑转移患者的无进展生存期(progression-free survival, PFS)明显优于非突变患者。此后, 吴一龙教授又在*Lung Cancer*发表文章^[19]^, 建议肺癌脑转移患者且仅有局部缓慢进展的患者可以考虑行局部治疗(放疗作为局部治疗的一种), 正式提出了肺癌治疗模式的问题。对于靶向治疗时代肺癌脑转移放疗的地位和参与方式提出了供业界参考的观点。此后, 肿瘤内科对于放疗的研究仍在进行。*Journal of Thoracic Oncology*杂志2016年发表TKI治疗基础上加或不加全脑放疗的文章, 结果提示放疗不能增加脑部局部控制率, 同时对于患者的生存也没有任何提升^[20]^。2017年, *Lancet Respiratory Medicine*发表研究^[21]^, 结果显示TKI与全脑放疗联合化疗相比, TKI疗效明显优于对照组。尽管该研究有其局限性, 两组对比资料治疗模式并不均衡, 但此文章与前期发表文章对于放疗的作用再次弱化。2016年, *Lancet*发表QUTARZ研究^[22]^的结论, 结果显示全脑放疗对于局部控制率和生存均没有获益, 尽管此项研究不牵涉TKI药物治疗, 但在TKI时代背景下, 对放疗的地位进一步弱化。放疗不仅不能提高局部控制率和生存, 还可能影响认知功能。诸多文献^[23, 24]^提示全脑放疗可能明显增加患者认知障碍风险, 这一问题又进一步加重了肿瘤内科学科选择脑部放疗的疑虑。

综上所述, 一系列的研究结果使肿瘤内科形成了其治疗模式, 内科观点是以症状和进展作为局部治疗介入的指征和标准, 没有症状的情况下, 给予局部放疗, 有可能增加患者的痛苦, 属于过度治疗。同时前期一些研究显示放疗对于局部控制率和患者生活质量没有提高, 同时又不能延长患者生存。因此, TKI时代对于没有症状的脑转移患者, 先应用药物处理, 待症状出现或者进展之后再行放疗, 成为肿瘤内科学科的主流见解和临床实践遵从的原则。

### 1.2 当前放疗视角

与内科学科的观点不同, 放疗学科也针对此问题做了一些研究, 但放疗学科迄今未有针对此问题的前瞻性研究结果。然而, 临床治疗的思路不能只关注随机对照试验的结果, 基于真实世界的临床回顾性研究也同样有很好的参考价值。同时, 放疗联合TKI能够互相协同, 提质增效有充分的理论基础^[25]^。同时前期研究^[26, 27]^也均显示两者联合毒性可以耐受。2016年, *Journal of Clinical Oncology*^[28]^发表了脑部放疗联合TKI对比单一TKI的文章, 结果显示放疗联合TKI能够明显延长患者生存。基于同样问题, 同一作者在2017年联合6个中心进行了多中心分析, 结果显示早行立体定向放疗组、早行全脑放射治疗(whole brain radiotherapy, WBRT)组和先用TKI组的中位总生存期分别为46个月、30个月和25个月, 2年总生存率分别为78%、62%和51%, 可见TKI基础上不管是加入全脑放疗还是立体定向放射外科(stereotactic radiosurgery, SRS), 结果都显示能够明显提高患者生存^[29]^。类似研究^[30, 31]^也表明, 基于TKI治疗的脑放疗可以显著改善患者的生存率和局部控制率。同时, 有研究^[32]^在专门针对脑转移分析集的探讨中发现, 在奥希替尼组, 如果近半年内患者做过脑部放疗的患者, 其有效率较无放疗患者也有提高的趋势(64% *vs* 34%)。在2018年世界肺癌大会(World Conference on Lung Cancer, WCLC), Enter研究^[33]^显示全脑放疗对比TKI联合脑部放疗的临床研究显示加入TKI没有使患者获益, 此阴性结果更是为TKI时代靶向治疗在脑部的效果泼了一盆冷水, 让人对TKI联合放疗的模式更加困惑。而针对寡转移晚期肿瘤患者局部进行放疗, 也取得了令人鼓舞效果, 其中这些局部治疗也包括了脑部转移患者的脑转移灶的放疗^[34]^。因此, 理论上来说, 不管是做哪一个部位的局部治疗, 除了增加局部控制率、缓解局部症状等目的, 局部治疗或多或少可以增加全身治疗的深度, 而治疗深度的增加可以带来患者生存的延长。同时, 也有研究^[35]^显示, 如果TKI耐药可能造成肿瘤的辐射抵抗, 降低脑转移放疗的疗效。可见, 与肿瘤内科系列研究相反, 放疗学科视角是对于局部病灶尽早处理, 可以尽可能地杀灭病灶, 增加治疗的深度, 进而达到延长患者生存的目的, 这一观点和模式也在肿瘤放疗学科影响广泛并在临床实践中得到贯彻。

尽管不同的研究表明放疗的疗效存在差异, 且不同研究的观点并不相同^[36, 37]^, 但总的来说, 与一系列有关内科肿瘤学的研究相反, 放疗学科的观点是尽早治疗局部病变, 尽早杀死它们, 增加治疗深度, 以达到延长患者生存时间的目的。这种观点和模式已广泛影响了肿瘤放射治疗的学科, 并已在临床实践中得到实施(图 1)。

**1:**
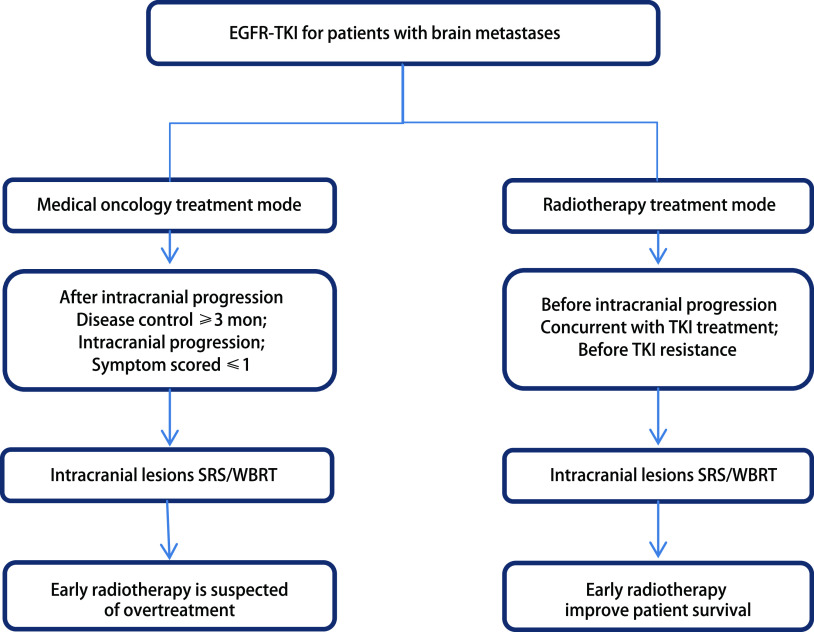
治疗模式之争图 Different treat mode. EGFR-TKI: epidermal growth factor receptor-tyrosine kinase inhibitor; SRS: stereotactic radiosurgery; WBRT: whole brain radiotherapy.

## 2 为什么形成了当前不同的治疗模式？

### 2.1 治疗模式之争历史中类似情况可寻

大家基于不同的学科理解形成了治疗模式之争(图 1), 但临床的真相只有一个, 学科发展要寻找的是真理不是立场。其实在医学发展中历史总有相似之处, 回顾小细胞肺癌的治疗历史^[38, 39]^, 当铂类药物取得近乎突破性的疗效时, 放疗地位的争论也随之而来, 但在进一步的研究中发现即便铂类治疗基础上, 也需要放疗的介入以便进一步提高患者的生存, 争论也随之平息。因此, *EGFR*敏感突变的NSCLC脑转移也需要进一步研究和更多临床数据的积累以便获得更加符合疾病治疗真相的模式。

### 2.2 即将消失的放疗研究

靶向治疗十几年来, 临床研究层出不穷, 但对于放射治疗学科来说, 靶向十年是失去的十年, 临床研究的数量与放疗在肿瘤治疗中的地位不符^[40]^。其中原因很多, 首先, 放疗学科是下游学科, 患者首诊一般不在放疗科, 因此, 药物企业主要推动肿瘤内科和外科进行临床试验, 进而放疗学科的数据较少; 其次, 靶向十几年来, 与药物治疗的巨大进步相比, 肿瘤治疗的多学科协作并没有同步发展, 进而影响放疗学科的参与度; 再次, 临床实验病例资料的收集过程中, 患者治疗环节太多不利于病例数据的总结随访, 其他学科出于这一考虑, 又进一步造成放疗学科与其他学科脱节。因此, 靶向治疗的十年, 伴随肿瘤内科治疗巨大突破的是放疗研究的相对减少。然而, 疾病的治疗与单纯的临床实验并不是一回事, 临床实验的偏颇也可能导致治疗的偏向, 药物治疗在带来肿瘤治疗巨大进步的同时, 也带来了学科发展的不平衡, 但临床实践需要患者获益的最大化。

### 2.3 让学科干自己的事情

分析肿瘤内科的临床试验, 对于脑部放疗探讨的重量级的几篇研究却不是放疗学科医生做的^[20, 21, 32]^。不同学科对于同一问题的理解都会有偏差, 肿瘤内科或者肿瘤外科对于放疗的理解也肯定有不全面之处, 进而在研究中可能影响患者入组, 观察随访, 得出的结论也可能与临床实际不符或者不尽相符。因此对于放疗的研究, 最好是由放疗科医生主导并进行设计, 以便得出更有价值和更接近真相的结论。

### 2.4 放疗学科临床研究需要更加个性化入组

脑转移患者放疗研究初始往往不分病种, 不论病理, 按照转移数目等进行入组^[41-46]^。而结合当前肺癌分子肿瘤学的发展, 临床实验需要放疗学科对于脑转移的研究不仅仅从放疗治疗角度出发, 还要结合药物治疗、对脑转移的认识和分子病理的迅速发展。同时按照基因突变、全身状况、分级预后评估(graded prognostic assessment, GPA)评分^[47, 48]^等综合因素进行更加个性化入组, 以便得出有价值、更符合临床实践的结论, 才能更好地指导临床治疗。

## 3 怎么解决当前治疗模式之争, 当前情况临床到底该如何选择？

最好的合作开展基于多学科的前瞻性临床研究, 而真相是需要时间探索和验证的, 但现实阶段学科认识的局限可以通过相对合理的治疗模式来规避。基于当前情况, 寻找患者和临床医生均可接受, 又有益于患者治疗的模式就需要多学科参与。而多学科综合治疗(multi-disciplinary treatment, MDT)模式的开展应该是最合理的选择。然而MDT需要制定可执行的、可监督的、能长期坚持的、临床参与各方又可及时获得反馈且最终又切实有利于患者的指导思想、操作流程、利益分配方式(图 2), 最终实现学科协同。避免学科偏颇、流于形式、不具备可执行性、不能坚持等MDT模式的开展, 进而避免给患者治疗造成的影响, 可能是当前对于患者较好的治疗模式^[49, 50]^。

**2:**
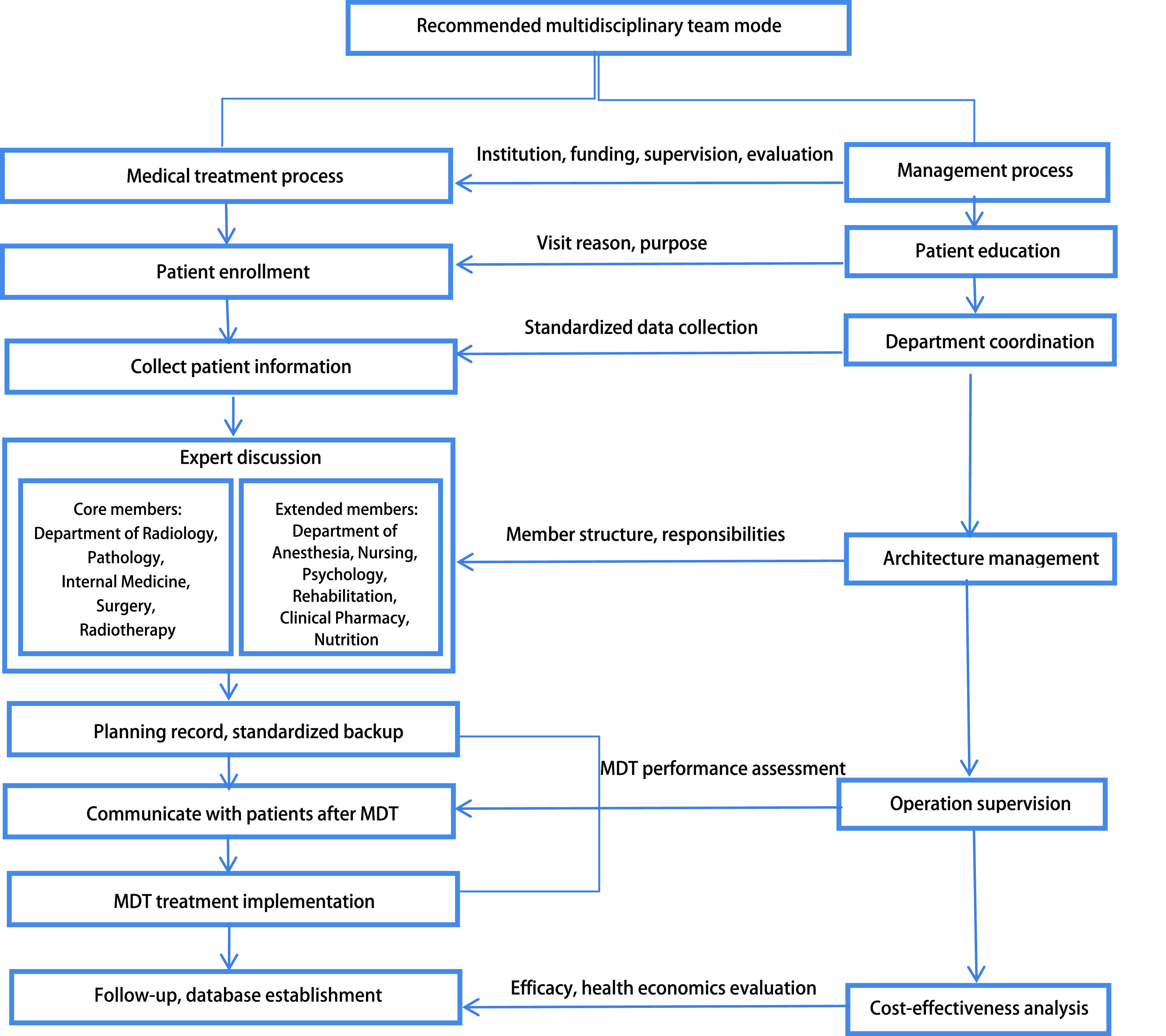
MDT模式图 MDT chart. MDT: multi-disciplinary treatment.

## 4 模式之争的未来是什么？

学科对于某一疾病的治疗模式^[51-53]^之争不是新问题, 伴随着疾病治疗手段的突破, 治疗模式之争更是容易出现, 其实这是学科发展的好事, 也是有益于患者的好事。任何学科之争都是阶段性的, 任何学科发展的不平衡也都是暂时的, 临床治疗之争是发展历程的一部分。针对学科之争, 树立解决问题并使患者获益的思想, 是每一个临床实验和临床医生的最终目标。当前采取合理的疾病治疗学科合作模式, 远期进一步进行临床实验以便探求真相, 治疗模式之争就一定能够避免当前学科认识局限, 并在未来真正推动学科发展, 以更好地造福患者, 我们也期待学科发展的光明未来。

## 5 结论

具有*EGFR*突变的NSCLC的脑转移是一个热门、困难且重点突出的研究课题, 也是肺癌治疗领域争议的焦点。学科之间的争议给临床医生的治疗选择带来了很多困惑。我们应该采取合理的疾病学科合作模式, 避免当前学科的局限性, 促进未来的发展, 使患者受益。
